# Effect of silica nanoparticles on clay swelling and aqueous stability of nanoparticle dispersions

**DOI:** 10.1007/s11051-013-2137-9

**Published:** 2013-12-04

**Authors:** Hieu Pham, Quoc P. Nguyen

**Affiliations:** Petroleum and Geosystems Engineering Department, The University of Texas at Austin, 200 E. Dean Keeton, Austin, TX 78712-1061 USA

**Keywords:** Nanoparticles, Montmorillonite clay, Polyethylene glycol (PEG), Swelling, Aqueous stability, Mobility, Coating, Oil recovery

## Abstract

The results of the effects of electrolyte type and concentration, nanoparticle concentration, pH, and temperature on the mobility and aqueous stability of polyethylene glycol (PEG)-coated silica nanoparticles are presented. Nanoparticle mobility was evaluated based on the ability to inhibit montmorillonite swelling in aqueous solutions through visual swelling tests, and the results were quantified in terms of the swelling index. The presence of PEG-coated silica nanoparticles was found to have a positive influence on the inhibition of clay swelling only in the presence of electrolytes. Quantification of nanoparticle stability in the presence of montmorillonite particles was achieved using ultraviolet–visible (UV–vis) spectrophotometry. At the highest concentration of montmorillonite dispersion studied, interaction between the dispersed montmorillonite particles and PEG-coated silica nanoparticles resulted in nanoparticle aggregation as indicated by increased turbidity and absorbance readings. Both nanoparticle concentration and montmorillonite dispersion concentration, in addition to the presence and concentration of NaCl, were found to strongly influence the stability of the mixture.

## Introduction

The use of nanotechnology has recently gained momentum in the oil and gas industry for its potential applications in enhanced oil recovery (EOR) and as nanosensors in hydrocarbon reservoirs (Sensoy et al. 2009; Amanullah and Al-Tahini 2009; Mokhatab et al. 2006; Krishnamoorti 2006; El-Diasty and Ragab 2013). Understanding of the transport of engineered nanoparticles in wellbores and hydrocarbon formations is necessary for successful application in the field. The mobility of engineered nanoparticles in natural formations is greatly influenced by the mineral and clay composition of the formation and by the dispersion stability of the nanoparticles. Subsurface environments typically vary over a wide range in regard to differences in electrolyte type and concentration, temperature, pH, and mineral composition.

In this work, we characterize the effects of a polyethylene glycol (PEG) coating on the transport and the aqueous stability of silica nanoparticles under conditions that are likely to be encountered in subsurface environments. Nanoparticle mobility was evaluated by studying the effects of electrolyte type (NaCl, KCl) and concentration, nanoparticle concentration, pH, and temperature on the inhibition of clay swelling. We also investigate the effects of finely dispersed montmorillonite particles on nanoparticle mobility through aqueous stability studies.

### Background

Nanoparticles can occur naturally or be synthesized from a variety of different materials and functionalized through surface modifications for a range of potential applications (Qhobosheane et al. 2001). Nanoparticles are typically in the scale of 1–100 nm, giving them a large surface area in addition to possessing unique mechanical, thermal, electronic, optical, magnetic, and chemical properties (El-Diasty and Ragab 2013; Krishnamoorti 2006). The emergence of interest in nanotechnology has led to several studies aimed at understanding the environmental impacts by investigating the transport, retention, and deposition of nanoparticles in saturated porous media. Studies on untreated metal oxide and C_60_ nanoparticles have found that the mobility is highly variable and dependent on the experimental setup (Ben-Moshe et al. 2010; Lecoanet et al. 2004; Wang et al. 2008). Studies by Ben-Moshe et al. (2010) and Lecoanet et al. (2004) suggest that nanoparticle mobility is dependent on numerous factors in natural systems, most notably humic acid and the ionic strength of the water. In addition, the larger surface area of nanoparticles increases the tendency for aggregation at low concentrations in aqueous solutions, which can alter the mobility of the nanoparticles (Hotze et al. 2010).

### Clay swelling and control

Clay swelling is widely considered to be a major cause for the formation of damage in hydrocarbon reservoirs and can greatly reduce nanoparticle mobility in porous media (Civan 2011; Amorim et al. 2007). During conventional oil production, the effects of clay swelling are most severe when incompatible injection fluids come into contact with swelling clays located on the rock matrices, resulting in greatly reduced formation permeability (Anderson et al. 2010; Hill et al. 2000).

Numerous studies have been devoted to characterize the crystal structure and hydration mechanisms of swelling clays. Clay minerals are classified as phyllosilciates, which form layered atomic structures consisting of two dimensional tetrahedral or octahedral sheets composed of silica and alumina. The arrangement of a single tetrahedral sheet joined to an octahedral sheet is called a 1:1 layer silicate structure, also referred to as T–O clay. If an additional tetrahedral sheet is found in the arrangement joined to the octahedral sheet, it is called a 2:1 layer silicate structure, also referred to as T–O–T clay (Anderson et al. 2010; Moore and Renyolds 1989).

Among studies in clay swelling, 2:1 smectite clays are often used as samples due to their high swelling capacity and will be the focus of our discussion. The smectite group encompasses several varieties of clay, notably montmorillonite because of its high swelling potential and frequency in hydrocarbon reservoirs. The layers in 2:1 layered silicates are overall negatively charged and are neutralized by the presence of cations in the interlayer space. The existing cations may be exchanged with other cations when the clay is exposed to cation-containing solutions. This property is called the cation exchange capacity (CEC) of the clay, which is defined as the maximum quantity of total cations that the clay is holding which is available for exchange with the solution for a given pH value (Moore and Renyolds 1989). Swelling occurs when polar molecules, such as water or organic molecules, adsorb onto the apical sites of the layers in the interplanar space. The expansion of the interlayer and swelling is thought to be primarily influenced by the type of exchangeable cations present in the aqueous solutions that come into contact with the clay (Luckham and Rossi 1999; Zhou 1995). For example, it is well established that swelling clays exposed to KCl-containing solutions exhibit lower swelling tendencies than NaCl-containing solutions.

Swelling occurs through two distinct processes depending upon the type and concentration of cations present in the aqueous solution in contact with the clay. Crystalline or microscopic swelling occurs under high brine concentrations or aqueous solutions high in divalent or multivalent ion concentrations. The initial entry of water forms surface complexes by hydrating the ions present in the interlayer space and forming hydrogen bonds to the clay surface oxygen atoms. Subsequent formation of additional monomolecular water layers on the clay surface can occur. Crystalline swelling results in relatively minimal swelling and the gross particle morphology is preserved. Osmotic swelling occurs in either dilute solutions or solutions containing large quantities of Na^+^ cations. The presence of Na^+^ cations results in the formation of an electric double layer on the surface of the clay mineral contributing to repulsive forces between the platelets (Luckham and Rossi 1999; Zhou 1995; Hensen and Smit 2002; Norrish 1954). Osmotic swelling results in large increase in the interlayer spacing and in clay volume by about 20 times (Norrish 1954). The difference between the two swelling processes can be determined by measuring the interplanar spacing of the clay in varying salt concentrations. Mohan and Fogler (1997) observed a discontinuity in the value of the interplanar spacing as the salt concentration was varied and defined the region as the critical salt concentration. Crystalline swelling occurs above the critical salt concentration while osmotic swelling occurs below this point.

There is great interest in identifying and investigating agents that reduce the degree of clay swelling. Polyols, such as glycols and glycerols, and recently amine based compounds have been established as effective shale inhibitors for the prevention of wellbore instability (Reid et al. 1995; Patel et al. 2007; Twynam and Caldwell 1994). Liu et al. (2004) evaluated the effectiveness of polyglycols in inhibition of montmorillonite swelling through linear swelling tests and particle size distribution experiments. The addition of PEG to KCl-containing solutions had little effect on the swelling height of the clay. However, results from size distribution curves of montmorillonite in KCl and PEG revealed an increase in the average particle size when compared to KCl alone. Carvalhido de Souza et al. (2010) observed that unmodified PEG was able to enter the interlayer region of the bentonite clay and reduce the clay water content. Hydrophobic modification of PEG improved swelling inhibition and reduction of water uptake by the clay.

### Stability of aqueous dispersion of engineered nanoparticles and clay particles

It is well established that the addition of free non-adsorbing polymers or smaller spherical particles can induce flocculation in a stable colloidal solution through the attractive depletion force (Jenkins and Snowden 1996; Mao et al. 1995). Recently, several groups have investigated the effects of the interaction between various adsorbing agents and clay suspensions. Takahashi et al. (2005) examined the dispersion stability of PE-G–clay hybrids in the context of drug delivery systems. At relatively low concentrations, acetal-PEG-b-PAMA was found to induce flocculation in the Laponite clay suspension. However, increased polymer concentrations were associated with enhanced stabilization of the dispersion, most likely attributed to the formation of a PEG brush shell layer shielding the negative charge of the clay nanocrystals. Pozzo and Walker (2004) studied the rheologic effects of the addition of poly(ethylene oxide) (PEO) to aqueous suspensions of Laponite. In the presence of PEO, the colloidal dispersion underwent reversible shear induced gelation. In general, the stability of the aqueous clay suspensions is influenced by the concentration of the added polymer and availability of the clay surface area for binding.

Similarly, the presence of fine migratory clay is thought to influence the aqueous stability of nanoparticles. Zhou et al. (2012) have shown that coagulation rates between montmorillonite and nanoparticles are greatly influenced by the pH and ionic strength of the solution. Jung et al. (2011) observed cross-linking of bentonite particles when iron oxide nanoparticle clay hydrids were added to the bentonite suspension. Al_2_O_3_–SiO_2_ nanoparticle clay hybrids were found to coagulate bentonite suspensions, however, only at low pH. Baird and Walz (2005) studied the effects of silica nanoparticles on the structure of aqueous kaolinite suspensions. Some degree of stabilization of the kaolinite suspension was observed only when the nanoparticles were added. Interestingly, the addition of both nanoparticles and salt caused the entire suspension to transition into a solid phase.

## Materials and methods

### Materials

The materials studied were aqueous dispersions of silica particles provided by 3 M. The mean diameters of the primary particles are 5 nm and have a modified surface with polyethylene glycol (PEG). Stock solution containing 21.2 % by weight 5 nm PEG-coated silica nanoparticles was diluted with deionized water to the desired concentration. Montmorillonite was purchased from Ward’s Natural Science Establishment, Inc. and dry sieved through 400 mesh sieves to obtain particle sizes less than 74 μm. The samples were dried at 110 °C overnight and stored in a desiccator jar prior to use. No further purification of the clay was performed. All experiments were performed at 25 °C unless otherwise stated. The pH of the solutions was adjusted by adding diluted sodium hydroxide solution (NaOH). Analytical grade quality NaCl and KCl were the inorganic salts used in the experiments.

### Montmorillonite swelling test

The protocol for visual swelling tests was adapted from American Society for Testing and Materials (ASTM)’s D5890-11 protocol. 0.1 g of montmorillonite was dispersed in previously prepared nanoparticle dispersions to obtain 5 g in total weight and sealed in glass vials. The swelling height of the montmorillonite was recorded using a caliper after a period of 3 days to allow the clay to sediment. The swelling index (SI) was calculated from the magnitude of the swelling height given by the formula SI = *h*/*h*_min_, where *h* is the height of the sample to the nearest hundredth of a millimeter and *h*_min_ is the minimal height of the montmorillonite sediment displaying maximum compression for a given set of samples.

### Aqueous stability test

To prepare the stock solution of fine clay mineral dispersion, montmorillonite was sieved through 400 mesh to obtain particle sizes less than 38 μm and dispersed in deionized water to achieve a water content of 100 g H_2_O per g clay. The resulting montmorillonite dispersion was allowed to settle for 24 h, and afterward manually agitated prior to centrifuging at 2,500 rpm for 45 min (IEC HN-SII Centrifuge, Damon/IEC Division). The supernatant was decanted and isolated while the precipitate was discarded. The supernatant was then subjected to centrifugation at 9,000 rpm for 15 min to obtain a clear dispersion (Eppendorf MiniSpin plus). The montmorillonite dispersion was added to previously prepared nanoparticle dispersions to achieve the desired concentrations. Since it is difficult to determine the final montmorillonite concentrations of these dispersions after centrifugation, the concentration of montmorillonite in the mixture is reported in terms of total mass of the dispersion added. The optical absorbance of the resulting mixture was analyzed using a Cary 50 ultraviolet–visible spectrophotometer (UV–vis) 1 h after mixing. The absorbance of a series of NaCl solutions within a concentration range from 0 to 1 wt% was measured to confirm the lack of NaCl absorbance in the ultraviolet region.

## Results and discussion

### Clay swelling with PEG coated silica nanoparticles

#### Effect of electrolytes

**Fig. 1:**
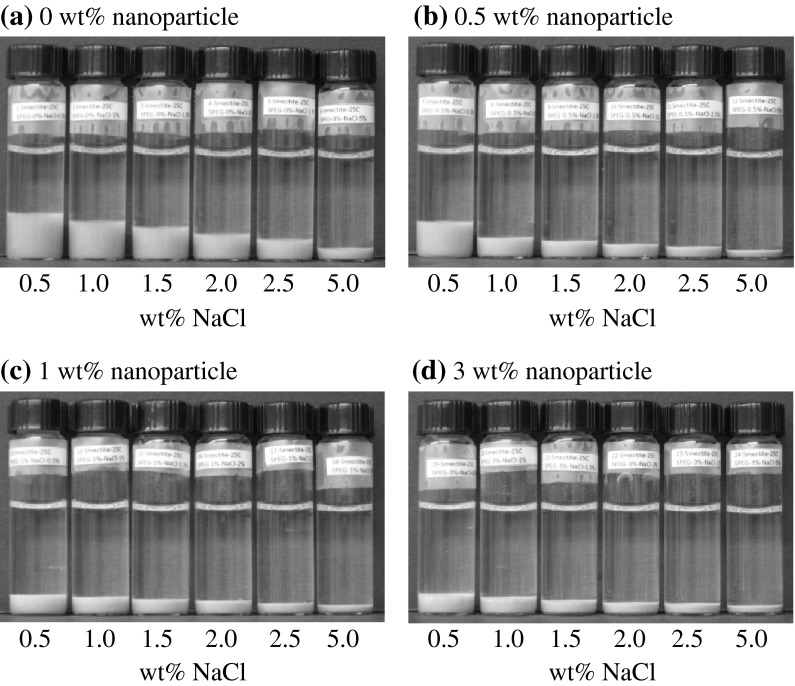
Photographs of sealed glass vials containing 0.1 g of montmorillonite in the presence of NaCl with: **a** deionized water only, **b** 0.5 wt%, **c** 1 wt%, and **d** 3 wt% nanoparticle concentrations

Montmorillonite is widely known to display decreased swelling potential in the presence of brine solutions such as NaCl and KCl. The macroscopic swelling of montmorillonite was visually observed in the presence of varying electrolyte concentrations. The effect of NaCl on clay swelling in the absence of nanoparticles is shown in Fig. 1a. The concentration of NaCl is increased from 0.5 to 5 wt% as is shown from left to right. As NaCl concentration increases, the volume of the clay sediment decreases. The same procedure was repeated in the presence of KCl and a similar trend was observed. However, the magnitude of clay swelling was considerably lower in the presence of KCl. This inhibition of clay swelling is thought to be due to the ability of KCl to compress the electrical double layer and decrease the electrostatic repulsion between the clay particles (Liu et al. 2004). Liu and Lu (2006) proposed that osmotic swelling in K-montmorillonite is inhibited energetically when the water content is higher than the range of the double-layer hydrate. Their study suggests that Na-smectites prefer expanded states (double layer, triple layer, and fully expanded) to a single-layer hydrate, while K-smectites prefer the single-layer state over the double-layer state. The K^+^ cations form coordinate structures with the oxygen atoms of the hexagonal ring situated on the clay surface and are effectively immobilized. The fixed K^+^ cations on either side of the interlayer space are able to share a water molecule in this region. The neutralization of the negative clay surface charge by K^+^ cations inhibits further swelling.

#### Effect of nanoparticles

**Fig. 2:**
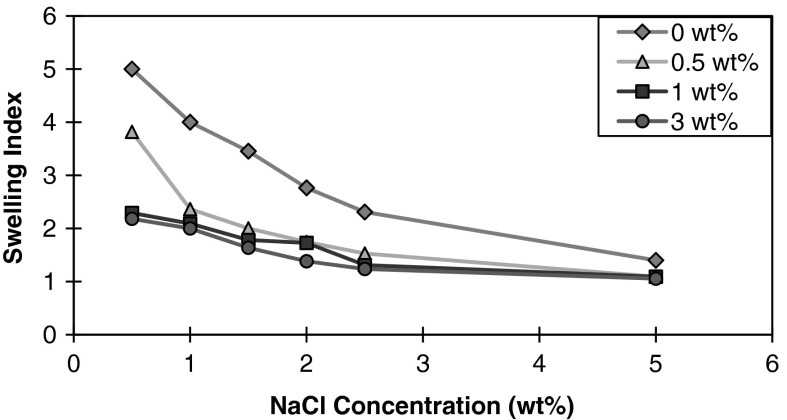
Swelling index of montmorillonite in the presence of varying nanoparticle concentrations as a function of NaCl concentration

Montmorillonite exposed to a series of solutions containing only nanoparticles in the absence of electrolytes resulted in a turbid mixture lacking a well-defined boundary between the clay mineral and aqueous solution. Addition of 5 nm PEG-coated silica nanoparticles to achieve 0.5 wt% in the presence of the brine solution is shown in Fig. 1b. When nanoparticles are present, a reduction in the magnitude of clay swelling was observed for all salt concentrations relative to dispersions containing NaCl only. The increasing nanoparticle concentration, as shown in Fig. 1, corresponds to a decrease in the magnitude of clay swelling. The swelling indices presented in Fig. 2 quantify the magnitude of swelling observed. All swelling in the presence of nanoparticles display approximately similar swelling indices at the maximum salt concentration studied (5 wt %). Nanoparticle concentrations of 1.5 and 3 wt% displayed similar swelling inhibition abilities throughout the salinity range. The most significant variation between swelling indices in the presence of nanoparticles was observed between 0.5 and 3 wt% nanoparticle concentrations at 0.5 wt% NaCl concentration by a factor of approximately 1.75 times. The next highest NaCl concentration at 1 wt% displayed a difference of only 1.18 between the two previously mentioned nanoparticle concentrations.

**Fig. 3:**
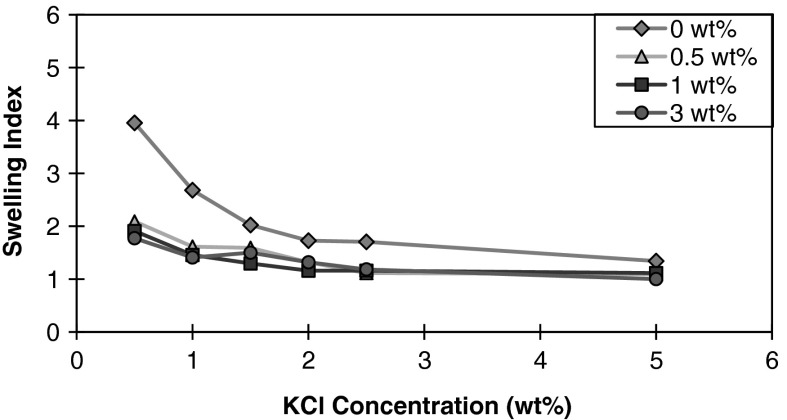
Swelling index of montmorillonite in the presence of varying nanoparticle concentrations as a function of KCl concentration

Montmorillonite exposed to both KCl and nanoparticles, as shown in Fig. 3, displays significantly less swelling compared to NaCl. The effects of the nanoparticle concentration do not follow a clear trend beyond 1 wt% KCl concentration compared to that of NaCl due to the near maximal compression of the clay sediment volume. Liu et al. (2004) suggested that polyglycols, such as PEG, in combination with KCl are effective in promoting clay particle aggregation and inhibition of clay swelling. The mechanism through which this process occurs is not well understood and is thought to be a result of water displacement through the adsorption of polyglycols onto the surfaces of clay minerals. K^+^ cations can act to stabilize the monolayer polyglycol–K^+^ complex in the interlayer space, which may explain its synergistic effect.

#### Effect of pH

**Fig. 4:**
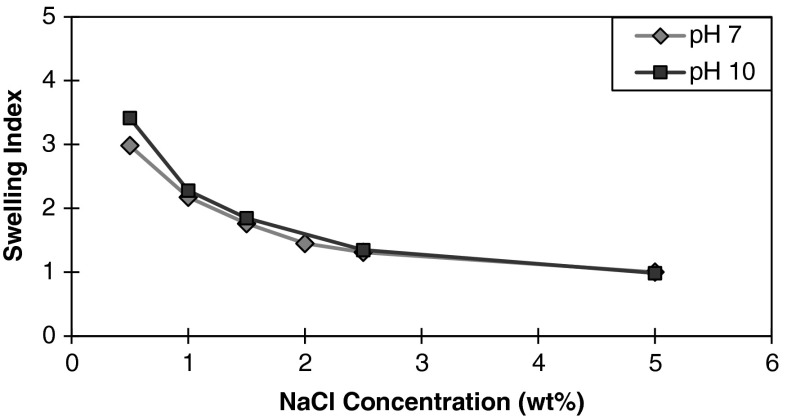
Swelling index of montmorillonite in the presence of 0.5 wt% nanoparticle concentration as a function of NaCl concentration for pH 7 and 10

In the absence of nanoparticles, there was no difference in the magnitude of clay swelling between pH 7 and 10. The permanent face charges of 2:1 silicate clays such as montmorillonite is primarily due to broken edges and isomorphic substitution, thus the CEC and swelling is independent of pH (Zhou et al. 2012). Figure 4 shows the swelling index for montmorillonite at pH 7 and pH 10 for a nanoparticle concentration of 0.5 wt % over a salinity range of 0.5 wt % to 5 wt %. At 0.5 wt % NaCl concentration, there is a noticeable difference between the swelling index at pH 7 and pH 10. This suggests that the difference in the swelling index is primarily due to the effects of the nanoparticles, rather than the CEC of the clay. Zhao et al. (1989) examined the adsorption rates and capacities of PEG onto montmorillonite clays. They found little difference in adsorbed amounts of PEG between pH 5 and 12 for Na-montmorillonite. The reduction in swelling inhibition effectiveness may be influenced more by nanoparticle stability than by adsorption. Metin et al. (2011) observed a decrease in the effective particle diameter for 25 nm bare surface silica nanoparticles at increasing pH values. In addition, the presence of salt can compress the electrical double of the nanoparticles and lower the energy barrier such that particle aggregation is driven primarily by kinetic energy. As the NaCl concentration increases, the swelling indices display similar values, suggesting that the effects of pH are gradually overwhelmed by the effects of electrolyte concentration.

#### Effect of temperature

**Fig. 5:**
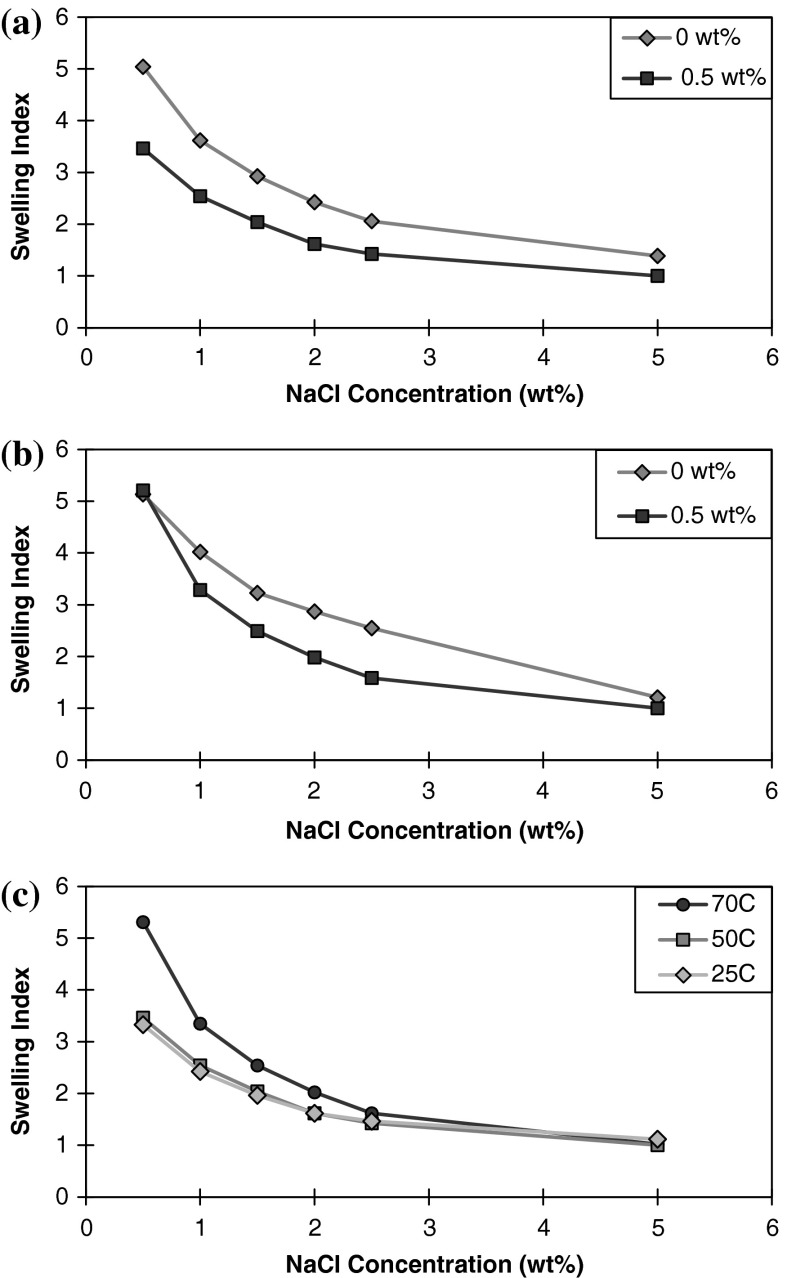
**a** Swelling index of montmorillonite in deionized water only and 0.5 wt% nanoparticle concentration as a function of NaCl concentration at 50 °C. **b** Swelling index of montmorillonite in deionized water only and 0.5 wt% nanoparticle concentration as a function of NaCl concentration at 70 °C. **c** Swelling index of montmorillonite in the presence of 0.5 wt% nanoparticle concentration as a function of NaCl concentration at 25, 50, and 70 °C

There is limited understanding of the effects of temperature on clay swelling in the presence of nanoparticles. Zhang et al. (1993) observed that the interlayer spacing was independent of temperature but that the *m*_*water*_ */m*_*clay*_ ratio decreased slightly with temperature at a given swelling pressure value. Zhou et al. (1997) also observed minimal change in interlayer spacing between 20 and 100 °C. Ishimori and Katsumi (2012) investigated the effect of temperature on the swelling capacity and barrier performance of bentonite in the context of geosynthetic clay liners. Free swell tests of bentonite were performed in NaCl concentrations ranging from 0.1 to 0.4 M. The free swell (mL/2 g) was observed to be greater for 60 °C as compared to 20 °C. Insufficiently swollen bentonites, such as those permeated with electrolytes, are more sensitive to temperature induced changes in the swelling volume than sufficiently swollen bentonites. Our results found that the swelling index was higher as the temperature increased in the absence of nanoparticles (Fig. 5a–b). The addition of nanoparticles resulted in a decrease in the swelling index for all temperatures studied. The swelling indices for montmorillonite exposed to a series of fixed 0.5 wt % nanoparticle concentration with different NaCl concentrations at elevated temperatures are shown in Fig. 5a Swelling index of montmorillonite in deionized water only and 0.5 wt % nanoparticle concentration as a function of NaCl concentration at 50 °C. As the temperature increases, the volume of clay sediment also increases for the same nanoparticle concentration. At 50 °C, the swelling index is initially slightly greater compared to 25 °C. The swelling index for 70 °C is dramatically greater by a factor of 1.59 compared to 25 °C at 0.5 wt % NaCl concentration.

### Aqueous stability of dispersions of PEG coated silica nanoparticles and montmorillonite particles

#### Effect of clay concentration

**Fig. 6:**
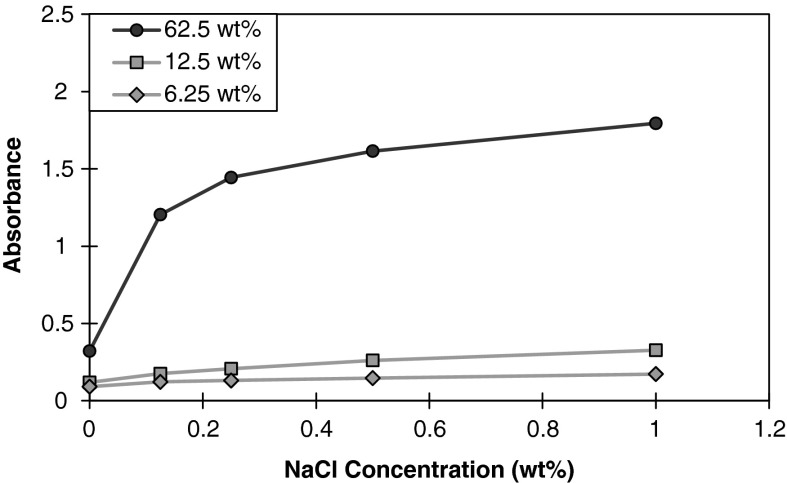
Absorbance at 300 nm of 6.25, 12.5, or 62.5 wt% montmorillonite dispersion in the presence of 1 wt% nanoparticle dispersion as a function of NaCl concentration

Three distinct phases of nanoparticle stability have been observed. A stable clear dispersed phase occurs below the critical salt concentration (CSC), while unstable turbid and separated phases occur above the CSC (Metin et al. 2011). The nanoparticles exhibited a stable clear dispersed phase as confirmed by stable absorbance measurements in the salinity range used throughout this experiment. The aqueous montmorillonite dispersion remained clear, although the absorbance readings showed slight increases towards higher NaCl concentrations. The effects of montmorillonite dispersion concentration in the presence of 1 wt % nanoparticle concentration are shown in Fig. 6. At the most dilute concentration studied, 6.25 wt %, there was no visual turbidity observed throughout the salinity range. Increasing the concentration to 12.5 wt % yielded similar results to the 6.25 wt % sample that were indiscernible to visual observations. The highest concentration of montmorillonite dispersion studied (62.5 wt %) yielded a significant difference in absorbance at 0.125 wt % NaCl and began to plateau as the NaCl concentration increased. Visual inspection of the mixture containing aqueous montmorillonite dispersion and nanoparticle dispersion was recorded to be clear, but became turbid immediately upon the addition of NaCl to the mixture which led to a sharp increase in absorbance. As stated previously, NaCl did not show any absorbance readings in the ultraviolet region. Derjaguin–Landau–Verwey–Overbeek (DLVO) theory describes the interaction between particle surfaces in terms of attractive Van der Waals and repulsive double layer potentials that are functions of distance between particles (Derjaguin and Landau 1941; Verwey and Overbeek 1948). Missana and Adell (2000) discussed the limitations of DLVO theory with regards to pH-dependent clay surface charges and uncertainties related to input parameters. Several studies have proposed modifications to account for the limitations of DLVO theory, although they all still predict a similar trend under low surface charge density conditions, i.e. increased aggregation due to greater compression of the diffuse layer under increasing ionic strength of the solution (Zhou et al. 2012; Jenkins et al. 2007). In pure water, montmorillonites can produce a homogeneous dispersion of hydrated nanocrystals, which is stabilized in part by the mutual repulsion of the interacting diffuse electrical double layers (Theng 2012). The presence of electrolytes such as NaCl compresses the electrical double layer to destabilize particle dispersion. The energy barrier of aggregation is sufficiently lowered such that kinetic energy governs the kinetics of particle aggregation. Recent findings suggest that agglomeration of the clay particles is thought to occur primarily through face–face contact, rather than edge–face and edge–edge (Zhou et al. 2012). The stability of these aggregates is influenced by the clay concentration of the suspension and the ions in the solution (Meunier 2005). The increasing concentration of montmorillonite particles provides greater opportunities for clay particle and nanoparticle collisions resulting in the increased turbidity observed. Zhou et al. (2012) studied the stability of montmorillonite particles in the presence of Ag and TiO_2_ nanoparticles. Their study suggests that the stability of a binary clay-nanoparticle system is dependent on the non-uniform distribution of the surface charge of clay platelets and additional surface contact interactions between clay particles and nanoparticles.

#### Effect of nanoparticle concentration

**Fig. 7:**
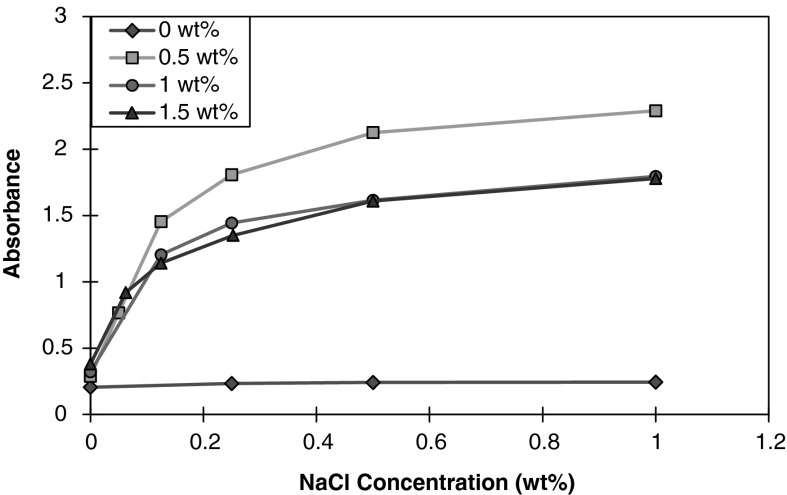
Absorbance at 300 nm of 62.5 wt% montmorillonite dispersion in the presence of varying nanoparticle concentrations as a function of NaCl concentration

Figure 7 shows the effects of silica nanoparticle concentration in the presence of aqueous montmorillonite dispersion at a fixed concentration of 62.5 wt%. The presence of nanoparticles at this concentration of montmorillonite dispersion greatly increases the turbidity of the mixture as the salt concentration is increased. At 0 wt% NaCl concentration, the absorbance increases with respect to increasing nanoparticle concentrations. As NaCl concentration exceeds 0.8 wt%, the absorbance curve for 0.5 wt% nanoparticle concentration begins to exceed both 1 and 1.5 wt% nanoparticle concentrations by significant values. The absorbance curves for 1 and 1.5 wt% nanoparticle concentration varies initially, but approach similar values beyond 0.5 wt% NaCl concentration. Takahashi et al. (2005) noticed a significant increase in turbidity upon the addition of PEG to a clay solution and proposed that the dispersion stability is dependent upon the polymer content. They observed flocculation of the clay suspension at low polymer content (PEG/clay ratio = 0.5), however, the solution became progressively clear with increasing polymer concentration. Zeta-potential measurements revealed that the magnitude of the surface charge of the PEG–clay complexes decreased as the polymer content increased until the surface charge was completely shielded at PEG/clay ratio = 2.5. They suggested that the shielding of the surface charge by PEG is a primary influence in the stability of the aqueous dispersion. The flocculation observed at low polymer content may be explained by the cross-linking of multiple clay nanocrystals by the polymer. As the polymer concentration increases, the PEG brush shell layer reduces the ionic interactions between negatively charged clay nanocrystals. The apparent lack of decrease in absorbance as nanoparticle concentration increased from 1 to 1.5 wt% suggests that the destabilization is limited by the availability of clay surface area beyond a certain nanoparticle/clay ratio.

#### Effect of temperature

**Fig. 8:**
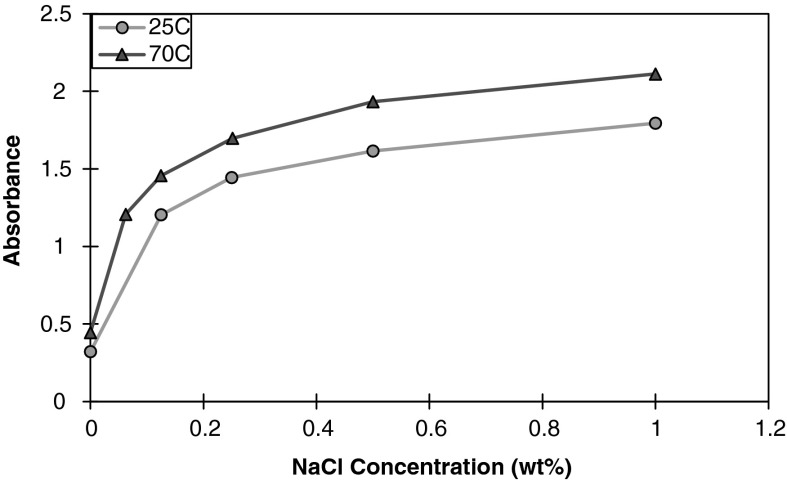
Absorbance at 300 nm of 62.5 wt% montmorillonite dispersion in the presence of 1 wt% nanoparticle concentration as a function of NaCl concentration at 25 and 70 °C

Temperature was increased to 70 °C and the absorbance was measured for a 1 wt% nanoparticle dispersion with an aqueous montmorillonite dispersion of 62.5 wt%. The results, as shown in Figs. 7 and 8, indicate that the turbidity increases at elevated temperatures. The stability of nanoparticles is known to be influenced by temperature, and the CSC is significantly reduced at elevated temperatures (Metin et al. 2011). The effects of Brownian motion and the average kinetic energy of the system are increased with increasing temperatures, resulting in more collisions that result in particle aggregation.

## Conclusions

We evaluated the mobility of 5 nm PEG-coated silica nanoparticles based on clay swelling inhibition behaviors and aqueous stability under parameters that include electrolyte type and concentration, nanoparticle concentration, temperature, and pH. The nanoparticles studied were found to inhibit clay swelling in the presence of NaCl and KCl in a synergistic manner. Samples containing only nanoparticles resulted in turbid mixtures lacking a well-defined boundary between the mineral and aqueous solution. The addition of nanoparticles to dispersions containing a fixed electrolyte concentration resulted in a greater reduction in the swelling index than is achieved by the electrolyte alone. KCl-containing dispersions were found to be more effective at inhibiting montmorillonite swelling than NaCl-containing dispersions. Across both electrolyte types, the swelling index generally decreased as the electrolyte concentration increased. The addition of nanoparticles resulted in reduced swelling indices for all nanoparticle concentrations as compared to electrolyte-only dispersions. However, the magnitude of reduction between swelling indices progressively diminished with increasing nanoparticle and electrolyte concentrations.

Increased pH had little influence on the swelling index for the nanoparticle concentration studied. Nanoparticles were shown to still be effective at inhibiting clay swelling at the highest temperature studied (70 °C) as compared to samples lacking in nanoparticles. For a fixed nanoparticle concentration, the swelling index was significantly greater at 70 °C as compared to 25 °C for the lower range of electrolyte concentration studied. Increasing the temperature from 25 to 70 °C in the presence of a high concentration of montmorillonite dispersion resulted in significant increases in turbidity. These observations suggest that the nanoparticle aggregation rate is increased at elevated temperatures, likely due to greater average kinetic energy and particle collisions.

Both nanoparticle and montmorillonite dispersion concentration were found to strongly influence the stability of the nanoparticles. Increasing the concentration of the added montmorillonite dispersion to the nanoparticle dispersion resulted in increased turbidity quantified by absorbance readings. The initial montmorillonite dispersion of high concentration and nanoparticle dispersions were clear, but became immediately turbid upon mixing in the presence of NaCl. The presence of NaCl was found to strongly influence particle aggregation within the mixture and compression of the electric double layer is thought to be the primary influence.

Addition of nanoparticles of increasing concentrations for a given high concentration of montmorillonite dispersion resulted in particle aggregation. Increased nanoparticle concentrations resulted in lowered absorbance readings compared to the lowest nanoparticle concentration studied. The effect is not yet fully understood, though observation suggests that the functionalized nanoparticles may confer some degree of stability to the mixture beyond a certain concentration.
